# International Scope of Emergency Ultrasound: Barriers in Applying Ultrasound to Guide Central Line Placement by Providers in Nairobi, Kenya

**DOI:** 10.1155/2018/7328465

**Published:** 2018-05-07

**Authors:** Fareen Zaver, Keith Boniface, Benjamin Wachira, Grace Wanjiku, Hamid Shokoohi

**Affiliations:** ^1^George Washington University, 2120 L Street NW, Suite 450, Washington, DC 2003, USA; ^2^Aga Khan University Hospital, Third Parklands Avenue, Limuru Road, Nairobi 00100, Kenya; ^3^Brown University, Box G-A1, Providence, RI 02912, USA

## Abstract

**Background:**

While ultrasound (US) use for internal jugular central venous catheter (CVC) placement is standard of care in North America, most developing countries have not adopted this practice. Previous surveys of North American physicians have identified lack of training and equipment availability as the most important barriers to the use of US.

**Objective:**

We sought to identify perceived barriers to the use of US to guide CVC insertion in a resource-constrained environment.

**Methods:**

Prior to an US-guided CVC placement training course conducted at the Aga Khan University Hospital in Nairobi, Kenya, physicians were asked to complete a survey to determine previous experience and perceived barriers. Survey responses were analyzed using summary statistics and the Rank-Sum test based on different specialty, gender, and previous US experience.

**Results:**

There were 23 physicians who completed the course and the survey. 52% (95% CI: 0.30–0.73) had put in >20 CVCs. 21.7% (95% CI: 0.08–0.44) of participants had previous US training, but none in the use of US for CVC insertion. The respondents expressed agreement with statements describing the ease of the use and improved success rate with US guidance. There was less agreement to statements describing the relative convenience and cost effectiveness of US CVC placement compared to the landmark technique. The main perceived barriers to utilization of US guidance included lack of training and limited availability of US equipment and sterile sheaths.

**Conclusion:**

Perceived barriers to US-guided CVC placement in our population closely mirrored those found among North American physicians, including lack of training and limited availability of US machines and equipment. These barriers have the potential to be addressed by targeted educational and administrative interventions.

## 1. Introduction

The frequency of usage of ultrasound (US) to guide central venous catheterization (CVC) placement has been varied among different institutions [1–4]. Prior studies have noted that cannulation success is correlated with the provider having a good working knowledge of vascular anatomy and using real-time sonographic guidance [5, 6].

Studies in anesthesia, the intensive care setting, and the emergency department have demonstrated that ultrasonographic guidance for the insertion of internal jugular vein central lines can lead to a decrease in complications, number of attempts, and cannulation time and improve overall success when compared to the landmark method [6–8]. In 2001, the Agency for Healthcare Research and Quality included real-time ultrasound guidance for CVC placement as an important patient safety goal [9].

Despite these guidelines, many centers have been slow to adopt US guidance as routine [10]. Multiple studies have evaluated the barriers to usage of US guidance in North America [1, 3, 4]. While ultrasound use for internal jugular CVC placement has become standard of care in both North America and the United Kingdom since approximately 2002, most developing world countries have not adopted this same standard of care and have been slow to adopt its use [11–13].

Henwood et al. suggested that the introduction of ultrasound may have a profound impact in a resource-limited setting [14]. Kenya is a lower-middle-income country in East Africa in which there are limited health resources and ultrasound trained personnel. After faculty at AKUHN in conjunction with the lead author conducted a needs assessment with emergency and critical care physicians in Nairobi, Kenya, it was determined that there was a great need to introduce real-time ultrasound guidance for CVC insertion and train providers in the technique.

Our main objective was to identify the barriers to the use of ultrasound to guide CVC insertion in our population. To our knowledge, this is the first study that looks at attitudes of providers in LMIC to the use of ultrasound for CVC placement.

## 2. Materials and Methods

### 2.1. Study Setting

The study was conducted at the Aga Khan University Hospital, Nairobi (AKUHN), where 20–40 CVCs are placed per month in the intensive care unit (ICU). These are inserted by critical care attending physicians, as well as the internal medicine, anesthesia, and surgery residents rotating through the ICU.

### 2.2. Study Design

This cross-sectional survey was administered to participants in an ultrasound training program conducted at AKUHN prior to the training program. The written survey was given out prior to this initial training and collected baseline characteristics regarding years of medical training, prior ultrasound training, and approximate number of prior central lines placed. It was followed by 12 questions that assessed the physicians' perspective of the advantages and disadvantages of the ultrasound-guided technique compared to the landmark technique (*supplementary online material *(available here)). The survey assessed the level of agreement to a particular statement including speed of placement, complications, infection control, and difficult placement situations according to a 5-point Likert scale. The final question was open-ended and asked about perceived barriers to using ultrasound guidance for CVC insertion. The study was approved by the IRB of the George Washington University, and all subjects provided informed consent.

### 2.3. Study Population

23 physicians participated in the ultrasound training and were enrolled in the study. This included every critical care attending physician at AKUHN as well as Kenyan internal medicine, anesthesia, and surgery residents certified to place CVCs who were rotating through the ICU during the three-month study period.

### 2.4. Data Analysis

The participants' demographic data and past experience with ultrasound and performing CVC placement were documented and reported as descriptive data. A survey analysis was performed of participants' perception of US-guided CVC on a 5-point Likert scale, ranging from strongly disagree through neutral to strongly agree. Survey responses were analyzed using summary statistics. The last question of the survey was an open-ended question eliciting perceived barriers to the use of ultrasound for CVC insertion. Responses were abstracted by the lead author and categorized. These categorizations were reviewed by the senior author and any disagreements were resolved via consensus. Sample size was determined by the number of participants in the training program. The data from this single-round survey were analyzed with limited reference to other information, using descriptive statistics. Tabulated responses were reported separately for each question asked, listing the number and percentage/proportion of answers in each category.

## 3. Results

There were 23 participants who completed the survey. The specialty distribution included critical care, anesthesia, emergency medicine, and internal medicine. The median number of years of practice was 6.5 years. Twelve participants (52%) had put in >20 central lines, eight participants (35%) put in between 1 and 20 CVCs, and three participants (13%) had never attempted CVC placement. Six participants (21.7%) had previous ultrasound training, but none had received any training on the use of ultrasound for CVC insertion (Table 1).

**Figure 1:**
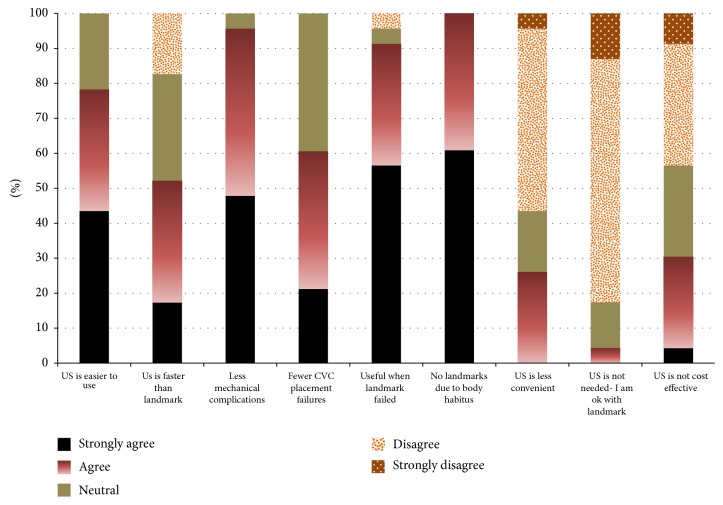
Distribution of participants' answers in comparing ultrasound guidance of CVC placement with that of traditional landmark technique.

Data were collected on the participants' perception of the “ease of use” of ultrasound for CVC placement. Both the physicians who had previously placed >20 CVCs (“experienced group”) and the physicians who had previously placed 20 or less CVCs (“inexperienced group”) agreed that it was easy to use (median of 4 out of 5 on Likert scale) (Figure 1). There was no significant effect of group experience (the mean ranks of the experienced group and nonexperienced group were 10.6 and 12.6, resp.; *p* value = 0.52). In addition, specialty had no significant effect on the survey responses.

Eleven out of 23 participants responded to the open-ended question eliciting perceived barriers, and responses clustered in two main areas of concern. The first barrier identified was a lack of training or comfort with the ultrasound machine. The second barrier was a limitation of resources to facilitate the performance of US CVC placement, including the availability of both the ultrasound machine and equipment to maintain sterility.

## 4. Discussion

AKUHN is a private, not-for-profit institution that provides tertiary care and advanced postgraduate medical training in East Africa. Anesthesiology and critical care attending physicians are all certified to place central venous catheters but had not previously received training in ultrasound guidance for CVC placement, a standard of care in North America and the United Kingdom. Our study demonstrated that the positive attitude toward ultrasound guidance among the physicians surveyed agreed with the objective findings of many randomized control trials and studies demonstrating that ultrasound was easier, faster, safer, and more successful in comparison to the landmark technique [6, 7, 10].

It is important to note that while some randomized trials and observational studies demonstrated that US guidance reduces the risks of complications and time to cannulation, these reductions varied depending on operator skill [11, 15]. Consistently, this was a common perceived barrier noted by many providers in the pretraining survey, lack of training and level of comfort with the ultrasound machine and cannulation technique. Buchanan et al. [1] recently published a similar survey study in the United States to determine barriers to US-guided CVC insertion, and they demonstrated that prior training in US-guided vascular access was the most important predictor of having a high comfort level with US guidance for CVC placement and a high rate of actual US use for CVC placement. The best outcomes in North America for increased use of US-guided CVC insertion have occurred when the introduction of ultrasound techniques is coordinated among departments and all levels of training [2, 5, 15, 16]. A cohesive approach making US machines readily accessible and supported by a strong educational program has reduced the learning curves and reluctance to adopt ultrasound [7].

Seventeen percent of the physicians in the present study reported that they feared that routine use of US-guided CVC insertion would lead to a decrease in skill with landmark technique, a large concern for those practicing in smaller centers without access to ultrasound. This fear could be addressed by demonstrating to learners that US-guided procedure can be a tutorial in applied anatomy, particularly for inexperienced proceduralists [6, 7], to improve understanding of the anatomy of the anterior neck, the impact of intrathoracic pressure on IJ caliber, and spacial relationship of the IJ vein to the carotid artery.

As training and lack of practice were identified as some of the greatest difficulties with US-guided CVC insertion, having regular simulation training allowing examination of normal anatomy on healthy volunteers, as well as visualizing abnormal anatomy such as a thrombus, arterial-venous overlap, and anatomy in obese or anatomically difficult patients, may decrease this perceived barrier.

Future studies should examine quality improvement measures that need to be in place to ensure a comprehensive review of all complications and skill and proficiency must be maintained. Logging all CVCs placed both under ultrasound and with landmark techniques and tracking their complications may determine if this skill actually improves health outcomes in a resource poor setting, either as part of a quality improvement project to ensure competency of providers, or as a research study. Results of this study will be useful for those designing US training programs in LMIC. In addition, patient-centered outcomes were not tracked in this survey study and should be the focus of future research projects.

## 5. Limitations

This survey was performed prior to the ultrasound training, and therefore the results are not necessarily generalizable to those who have already received formal training. Additionally, the Aga Khan University Hospital is a relatively well-resourced private hospital, which limits the generalizability of the data to public hospitals in the region, which have fewer resources. Additionally, AKUHN is a teaching facility and thus the results may not be applicable to nonteaching hospitals. The survey was also self-reported and thus unknown bias or errors may have resulted.

## 6. Conclusions

Perceived barriers among Kenyan physicians to the use of ultrasound guidance for the placement of CVCs mirror those found in the United States and include lack of training in and decreased comfort level with the technique, as well as not having the necessary ultrasound equipment and sterile sheaths. Barriers pertaining to training can be addressed with educational interventions, while barriers related to limited resources can be overcome with a coordinated effort among hospital administrators, equipment manufacturers, and funders.

## Figures and Tables

**Table 1:** The participants' demographic data and past experience with ultrasound and performing CVC placement (*n* = 23).

Variable	Number (% [95% CI])
*Gender*	
Female	10
Male	13
*PG training*	
1	4
2	6
3	2
>=4	5
Nonspecified	6
*Year(s) in practice*	
<=5 years	16
>5 years	7
*Specialty*	
Internal medicine	6
Critical care	5
Anesthesia	5
Surgery/surgical subspecialties	4
Emergency medicine	2
Others	1
*Number of CVCs placed*	
>20	12 (52% [30–73])
1–20	8 (35% [15–54])
None	3 (13% [0–26])
*Prior ultrasound training*	6
*Prior use of ultrasound for CVC placement*	None

## Supplementary Materials

Supplementary Materials Supplementary File: a written survey of practices for the use of ultrasound for CVC placement, representing participants' perspectives on the benefits of ultrasound-guided CVC placement as compared to landmark technique.
