# Supplementary material for: International Scope of Emergency Ultrasound: Barriers in Applying Ultrasound to Guide Central Line Placement by Providers in Nairobi, Kenya
Source: Emerg Med Int. 2018 May 7;2018:7328465. doi: 10.1155/2018/7328465 (PMC5964574; doi:10.1155/2018/7328465)
Supplement: Supplementary Materials — Supplementary File: a written survey of practices for the use of ultrasound for CVC placement, representing participants' perspectives on the benefits of ultrasound-guided CVC placement as compared to landmark technique. [file 7328465.f1.pdf]

Central Venous Catheter Insertion Survey:

Year of Training / Number of years in practice: _____ / ______

Gender: MALE FEMALE

Specialty: CRITICAL CARE ANESTHESIA EMERGENCY MEDICINE OTHER

Number of prior central lines placed (circle one): 1 1-5 5-9 10-20 >20

Estimate the percentage of those patients in whom you were successful:

0-20% 21-40% 41-60% 61-80% 81-100%

When using the landmark technique, how often do you confirm venous cannulation before inserting the dilator (ABG, pressure transducer)?

0-20% 21-40% 41-60% 61-80% 81-100%

Any prior ultrasound training: YES NO

If yes please specify:

What type of ultrasound (catheter insertion, FAST exams, cardiac exams, etc):

Level of comfort with ultrasound (circle one):

VERY SOMEWHAT NOT AT ALL N/A

Have you used the ultrasound machine for central venous catheter insertion before:

YES NO

For each of the following please indicate your level of agreement:

When compared to the landmark method, ultrasound guided central line placement:

Is easier to use

Strongly Agree Agree Neutral Disagree Strongly disagree

Is faster

Strongly Agree Agree Neutral Disagree Strongly disagree

Reduces complications such as pneumothorax, arterial puncture (etc)

Strongly Agree Agree Neutral Disagree Strongly disagree

Reduces infectious complications

Strongly Agree Agree Neutral Disagree Strongly disagree

Results in fewer placement failures

Strongly Agree Agree Neutral Disagree Strongly disagree

Is useful when the landmark method has been unsuccessful

Strongly Agree Agree Neutral Disagree Strongly disagree

Is useful when a patient lacks landmarks due to body habitus

Strongly Agree Agree Neutral Disagree Strongly disagree

Is less convenient

Strongly Agree Agree Neutral Disagree Strongly disagree

Is not needed (I am successful with the landmark method)

Strongly Agree Agree Neutral Disagree Strongly disagree

Will result in a loss of my skills

Strongly Agree Agree Neutral Disagree Strongly disagree

Is not cost effective

Strongly Agree Agree Neutral Disagree Strongly disagree

Is difficult to use due to resources

Strongly Agree Agree Neutral Disagree Strongly disagree

Do you perceive any difficulties in using ultrasound for central venous catheter insertion:

YES NO

If yes, what are these specific barriers?
